# The Food Anti-Microbials β-Phenylethylamine (-HCl) and Ethyl Acetoacetate Do Not Change during the Heating Process

**DOI:** 10.3390/antibiotics10040418

**Published:** 2021-04-10

**Authors:** Shelley M. Horne, Angel Ugrinov, Birgit M. Prüβ

**Affiliations:** 1 Department of Microbiological Sciences, North Dakota State University, Fargo, ND 58108, USA; sm.horne@ndsu.edu; 2 Department of Chemistry and Biochemistry, North Dakota State University, Fargo, ND 58108, USA; angel.ugrinov@ndsu.edu

**Keywords:** food anti-microbial, β-phenylethylamine, ethyl acetoacetate, gas chromatography, mass spectrometry, minimal inhibitory concentration, minimal bactericidal concentration

## Abstract

β-Phenylethylamine hydrochloride (PEA-HCl) and ethyl acetoacetate (EAA) are anti-microbials with applications in food processing. As food anti-microbials, the compounds will have to withstand the cooking process without changing to toxic compounds. With this Communication, we address the question of whether PEA and EAA are altered when heated to 73.9 °C or 93.3 °C. A combination of gas chromatography and mass spectrometry was used to analyze solutions of PEA(-HCl) or EAA in beef broth or water. In addition, the anti-microbial activity of PEA-HCl and EAA was compared between heated and unheated samples at a range of concentrations. The gas chromatograms of PEA(-HCl) and EAA showed one peak at early retention times that did not differ between the heated and unheated samples. The mass spectra for PEA and EAA were near identical to those from a spectral database and did not show any differences between the heated and unheated samples. We conclude that PEA(-HCl) and EAA formed pure solutions and were not altered during the heating process. In addition, the anti-microbial activity of PEA-HCl and EAA did not change after the heating of the compounds. Regardless of temperature, the minimal inhibitory concentrations (MICs) for PEA-HCl were 20.75 mmol mL^−1^ for *Escherichia coli* and *Salmonella enterica* serotype Typhimurium. For EAA, the MICs were 23.4 mmol mL^−1^ for *E. coli* and 15.6 mmol mL^−1^ for *S. enterica*.

## 1. Introduction

Previous research by our own laboratory identified β-phenylethylamine-HCl (PEA-HCl) and ethyl acetoacetate (EAA) as novel anti-microbials with a broad range of effectiveness against numerous bacterial pathogens and in several applications. PEA-HCl and acetoacetate (AAA) were originally discovered as anti-microbials against *E. coli* O157:H7 in a screen of 95 carbon and 95 nitrogen sources [1]. A potential medical application of PEA-HCl was its use as a liquid flush for silicone tubing in an experiment that was modeled after antibiotic lock treatment [2]. A research group in Zürich, Switzerland described PEA-HCl as an organic food additive that inhibits *Listeria monocytogenes* in ready-to-eat foods [3]. EAA, which is chemically similar to AAA and more cost-effective, inhibited growth and biofilm formation by *Yersinia enterocolitica*, *Serratia marcescens*, and *Cronobacter sakazakii* [4]. In a food safety application, both PEA-HCl and EAA were used as treatments for ground beef, where they reduced naturally occurring spoilage bacteria [5]. Research on the effectiveness of PEA-HCl and EAA at inhibiting bacteria when used as a processing aid for chicken is in progress. A patent on EAA as a novel anti-microbial has been submitted and is pending [6].

There is evidence that neither PEA-HCl nor EAA should be toxic when used as treatments for food and certainly not when used as a processing aid, where a smaller amount of the anti-microbial is left on the food. PEA-HCl is described by the World Health Organization (www.who.org; accessed on 9 April 2021) as a flavoring agent of no safety concern and is generally regarded as safe (GRAS status) (ASP 1213; 000064-04-0). Walmart (www.walmart.com) and Amazon (www.amazon.com) sell PEA-HCl in capsules of 1.500 mg and boxes of 500 g for the purpose of aiding weight loss and improving mood. EAA has FDA approval as a food additive under 21CFR172.515 and is used for flavoring under Flavis No. 9.402.

However, the use of PEA-HCl and EAA in some food applications (e.g., meat) requires cooking. This raises the question of whether the cooking process may convert PEA-HCl and/or EAA to some other compound that could potentially be toxic. As a first example of anti-microbial degradation during the heating process, degradation in boiled milk was determined by a combination of liquid chromatography (LC) followed by mass spectrometry (MS). Cefoperazone was the least stable and cloxacillin the most heat-stable substance, with 78.3% and 9.6% degradation in 300 s, respectively [7]. In this study, degradation was desired, as the most undesired substance in the milk is the original anti-microbial. In a second example, the heat stability of amphenicols such as chloramphenicol was dependent on the matrix that the anti-microbial had been dissolved in. Heat degradation was accelerated in soybean sauce relative to water and not protected in meat [8].

We used a combination of gas chromatography (GC) and MS to determine whether PEA(-HCl) or EAA change after heat treatment at 73.9 °C and 93.3 °C in beef broth. There was no difference in the gas chromatograms and mass spectra between the heated and unheated PEA(-HCl) or EAA samples under any of the many conditions tested. Likewise, the anti-microbial activities of PEA-HCl and EAA against two food pathogens, *E. coli* O157:H7 and *Salmonella enterica* serovar Typhimurium, did not change and the minimal inhibitory concentrations (MICs) and minimal bactericidal concentrations (MBCs) were the same for heated and unheated samples of PEA-HCl and EAA. We conclude that PEA(-HCl) and EAA did not change during heating in beef broth. 

## 2. Results

### 2.1. GC–MS Analysis: PEA(-HCl) Did Not Change during the Heating Process in Beef Broth

**Figure 1:**
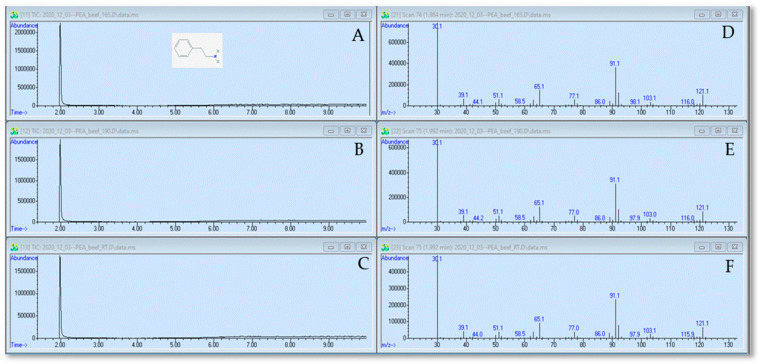
GC chromatograms of PEA and MS spectra of the 2 min peaks. Panel (**A**–**C**), chromatograms for PEA unheated (Panel (**A**)), PEA heated to 73.9 °C (Panel (**B**)), and PEA heated to 93.3 °C (Panel (**C**)). The *x*-axis is retention time in min. Panels (**D**–**F)**, spectra of the 2 min peaks from the respective GC. The *x*-axis is *m/z*, where *m* is the mass and *z* is the charge. The PEA molecule integrated in Panel A was taken from PubChem (https://pubchem.ncbi.nlm.nih.gov/compound/).

Three samples were analyzed by GC–MS: 83 µmol mL^−1^ PEA in 1% beef broth unheated, heated to 73.9 °C, and heated to 93.3 °C. The chromatograms from the GC are demonstrated in Figure 1A,C; each of the chromatograms showed a sharp peak at 2 min retention time and the three chromatograms were very similar to one another.

The eluates from the 2 min peaks were further analyzed with MS (Figure 1D,F); the MS spectra for the three samples were nearly identical. The five predominant peaks were at 30 atomic mass units (amu), 65 amu, 91 amu, 92 amu, and 121 amu. Note that 121 g mol^−1^ is the molecular weight of PEA. The result was compared with MS analysis of PEA from the Spectral Database for Organic Compounds (SDBS) (Table 1).

The mass spectrum from our experimental PEA is practically identical to the SDBS pattern (Table 1). In both cases, the primary peak is at 30 *m/z*, while the second most intense peak is at 91 *m/z*. The near identity between our experimental and the SDBS spectra for PEA indicates that the 2 min peak from the GC contains pure PEA. The qualitative comparison of the chromatograms from the three samples (Figure 1A–C) leads to the observation that heating did not yield new peaks for PEA, indicating that PEA did not produce novel compounds during heating in beef broth.

To make the statement that the quantity of PEA was not reduced by heating the compounds, we performed a quantitative analysis of the 2 min peaks from the GC. The areas of the peaks were determined by integrations from four replicate experiments and were calculated to PEA concentration in µmol mL^−1^ with a calibration curve. Averages and standard deviations were 93 µmol mL^−1^ ± 5.4% for the unheated sample, 100 µmol mL^−1^ ± 3.3% for the 73.9 °C sample, and 98 µmol mL^−1^ ± 3.9% for the 93.3 °C sample. Interestingly, there appeared to be a small increase of 8.5% for the 73.9 °C sample and 6.9% for the 93.3 °C sample. In any case, there was no decrease in the PEA concentration due to the heating process. We conclude that the amount of PEA within the 121.1 amu peak did not decrease during heating.

To determine whether heat degradation of the anti-microbial may be dependent on the concentration of beef broth, three identical solutions of PEA-HCl in 5% beef broth were heat-treated as described above and analyzed with GC–MS. Across three replicate experiments, the PEA-HCl concentrations were calculated as follows: 640 µmol mL^−1^ ± 8.2% for the unheated sample, 641 µmol mL^−1^ ± 7.3% for the 73.9 °C sample, and 652 µmol mL^−1^ ± 4% for the 93.3 °C sample. Differences in PEA concentration between samples equaled an increase of 0.16% for the 73.9 °C sample and 1.9% for the 93.3 °C sample. Increasing the concentration of beef broth did not cause heat degradation of PEA-HCl.

### 2.2. GC–MS Analysis: EAA Did Not Change during the Heating Process

**Figure 2:**
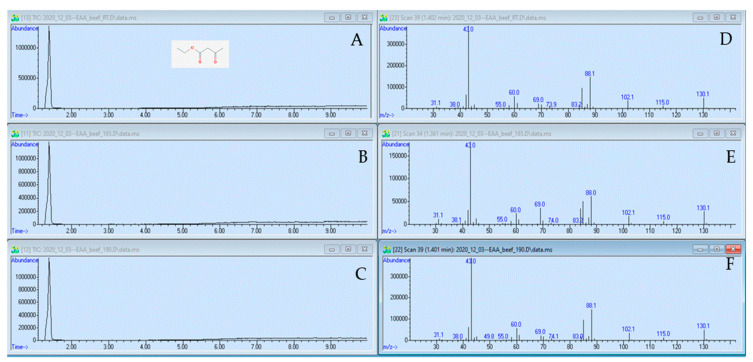
Chromatograms of EAA and spectra of the 1 min peaks. Panel A to C, chromatograms for EAA unheated (Panel (**A**)), EAA heated to 73.9 °C (Panel (**B**)), and EAA heated to 93.3 °C (Panel (**C**)). The *x*-axis is retention time in min. Panels (**D**–**F**), spectra of the 1 min peaks from the respective GC. The *x*-axis is *m/z*. The EAA molecule integrated in Panel (**A**) was taken from PubChem (https://pubchem.ncbi.nlm.nih.gov/compound/).

EAA was analyzed in three samples: 77 µmol mL^−1^ EAA in 1% beef broth unheated, heated to 73.9 °C, and heated to 93.3 °C. The GC chromatograms and MS spectra are shown in Figure 2. The MS spectra were compared with MS analysis of EAA from SDBS (Table 2).

In both the experimental EAA sample and the SDBS mass spectrum for EAA, the main MS peak was at 43 amu. The remaining mass fragments were identical between the three samples, with minor differences in peak intensities. The large degree of similarity between our experimental and the SDBS spectrum for EAA indicates that our 1 min peak from the GC contains pure EAA. The qualitative similarity of the GC chromatograms from the three samples (Figure 2A–C) implies that EAA did not produce novel compounds during heating that would show as a peak in the GC.

A quantitative analysis was performed on the GC–MS data to compare the amount of EAA in the 1 min peaks. The area of the peak was determined by integrations from four replicate experiments and was used to calculate EAA concentration as µmol mL^−1^ with the help of a calibration curve. Averages and standard deviations were 97 µmol mL^−1^ ± 4.34% for the unheated sample, 93 µmol mL^−1^ ± 7.75% for the 73.9 °C sample, and 91 µmol mL^−1^ ± 2.15 for the 93.3 °C sample. The difference in EAA concentration between the unheated and the 73.9 °C sample is a decrease of 4%, which is less than the standard deviations for each of these samples. The difference in EAA concentration between the unheated and the 93.3 °C sample equals a 6.3% reduction by the heating process. While this is still less than the standard deviation of the 73.9 °C sample, the possibility cannot be excluded that this experiment shows a small reduction in the EAA concentration due to heating of the sample to 93.3 °C.

To determine whether heat degradation of EAA may be dependent on the beef broth concentration, EAA was dissolved in 5% beef broth, heat-treated, and analyzed with GC–MS. The EAA concentrations in the main peaks were determined as follows: 290 µmol mL^−1^ ± 4.7% for the unheated sample, 296 µmol mL^−1^ ± 4.2% for the 73.9 °C sample, and 289 µmol mL^−1^ ± 4.9 for the 93.3 °C sample. Differences in EAA concentration between samples equaled an increase of 2.1% for the 73.9 °C sample and a reduction of 0.6% for the 93.3 °C sample. Both these changes are within the standard deviations for the respective samples. It does not appear that a higher concentration of beef broth leads to an increase in the heat degradation of EAA.

### 2.3. Heating Did Not Change the Antimicrobial Activities of PEA-HCl and EAA

To compare the antimicrobial activities of PEA-HCl and EAA between the different heat treatments, we determined minimal inhibitory concentrations (MIC) and minimal bactericidal concentrations (MBCs). Bacteria of *E. coli* O157:H7 and *S. enterica* were used to determine the anti-microbial activity of unheated, 73.9 °C heated, and 93.3 °C heated PEA-HCl and EAA. Bacteria were grown for 24 h at 30 °C in beef broth at increasing concentrations of the anti-microbials. 

**Figure 3:**
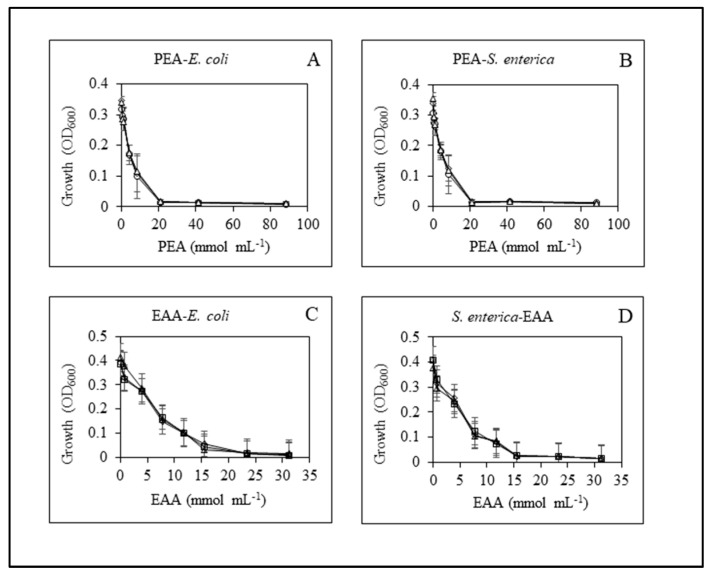
Anti-microbial activities of unheated, 73.9 °C heated, and 93.3 °C heated PEA-HCl (Panel (**A**,**B**)) and EAA (Panel (**C**,**D**)). *E. coli* and *S. enterica* were used as reference strains. Squares, unheated samples; diamonds, 73.9 °C samples; triangles, 93.3 °C samples.

Growth of *E. coli* was determined as OD_600_ and declined with increasing concentrations of PEA-HCl (Figure 3A). For unheated, 73.9 °C heated, and 93.3 °C heated PEA-HCl, the MIC was 20.75 mmol mL^−1^. The growth pattern for *S. enterica* was similar (Figure 3B). The MICs for unheated, 73.9 °C heated, and 93.3 °C heated PEA-HCl were also 20.75 mmol mL^−1^. EAA prevented growth at a similar range of concentrations. We consider 23.4 mmol mL^−1^ the MIC for EAA to reduce the growth of *E. coli*. For the growth of *S. enterica*, the MIC for EAA was lower, at 15.6 mmol mL^−1^.

MBCs were determined from live bacterial counts at concentrations in the vicinity of the respective MIC. For unheated, 73.9 °C heated, and 93.3 °C heated PEA-HCl, the MBC was 20.75 mmol mL^−1^ for both *E. coli* and *S. enterica*. This concentration is identical to the MIC.

For EAA, there was a range of concentrations that yielded very similar percent reductions, which made the MBC determination more difficult. The data are presented in Table 3. For unheated, 73.9 °C heated, and 93.3 °C heated EAA, the first concentration at which a 99.9% reduction was accomplished was 31.2 mmol mL^−1^ for *E. coli*. We consider this concentration the MBC. While this MBC is higher than the MIC, the percent reductions in live bacterial counts were already between 99.68% and 99.85% for samples of all heat treatments at the MIC concentration of 23.4 mmol mL^−1^. It is possible that the difference between MIC and MBC is due to the definition (cutoff) of MIC and MBC rather than a real difference between the ability of the anti-microbial to inhibit bacterial growth and actually kill the bacteria. Using the same cutoff of 99.9% for the MBC for *S. enterica*, the MBC for the unheated and the 73.9 °C samples would be 31.2 mmol mL^−1^ as well. Intriguingly, for the 93.3 °C sample, the MBC would be 15.6 mmol mL^−1^, which is identical to the MIC and lower than that for the unheated sample. However, if we look at the percent reduction across the entire experiment, we believe that there is no noticeable difference in the anti-microbial activity between the three samples. We are hesitant to define an MBC for EAA and *S. enterica* with this rather large range of concentrations that yields similar percent reductions. Nevertheless, heating the samples does not increase the MBC or decrease the effectiveness of EAA as an anti-microbial for both *E. coli* and *S. enterica*. Heating either PEA-HCl or EAA up to 93.3 °C did not change the anti-microbial activities of the compounds for the two common food pathogens tested.

## 3. Discussion

We were unable to identify any differences in the GC chromatograms and MS spectra between heated and unheated samples of PEA(-HCl) and EAA, dissolved in beef broth. Furthermore, the mass spectra for PEA and EAA were nearly identical to those obtained from SDBS for the same compounds. We conclude that the solutions of PEA(-HCl) and EAA were pure and that our anti-microbial compounds did not change during the heating process in a medium that was similar to the beef meat environment and consisted of mixed amino acids and other organic as well as inorganic compounds. To ensure that the anti-microbial activities of PEA-HCl and EAA were not affected during heating, MIC and MBC were determined for one *E. coli* O157:H7 and one *S. enterica* serotype Typhimurium strain. For both bacteria and both anti-microbials, the anti-microbial activities were very similar to identical for heated and unheated samples.

The question of whether PEA (-HCl), and/or EAA might change during the heating process arose during discussions on the toxicity of these compounds. For PEA, a distinction needs to be made between PEA and its hydrochloride, PEA-HCl. When synthesized in a living being, usually in small amounts, PEA is the decarboxylation product of phenylalanine. It can be found in animals [9], plants [10], and in the human brain [11,12]. In the brain, PEA serves as a neurotransmitter via a signal transduction pathway that involves the trace amine-associated receptor 1 (TAAR1) [13,14]. On the vascular system, PEA acts as a vasoconstrictant [15]. The turnover of PEA in the human body is quick and involves the enzyme monoamine oxidase [16]. PEA can be found in trace amounts in chocolate, as a result of the thermal processing of cocoa [17,18]. It can be produced by food bacteria, but tyramine is the trace amine that causes the food safety concern in this context [19]. Interestingly, applications that involve PEA use the hydrochloride form, PEA-HCl, in much larger concentrations. This includes food applications [3,5], clinical applications [2], as well as using PEA-HCl as a dietary supplement (Amazon, Walmart, Swanson Health). Additionally, GRAS status was awarded by the FDA to PEA-HCl and not PEA. For our GC–MS experiments, we used both PEA and PEA-HCl. To determine anti-microbial activities, we used the actual anti-microbial, PEA-HCl.

EAA is the ethyl ester of AAA. It is used as a flavoring ingredient for food (Flavis No 9.402) and the FDA approved it as a food additive under 21CFR172.515, albeit at an unidentified concentration. A number of older studies indicate that (i) feeding rats with up to 300 mg/kg body weight of EAA did not result in any health defects or changes in hematology, serum, urine, or renal function [20]; (ii) EAA caused moderate but reversible bovine eye irritation [21], and (iii) EAA exhibited no mutagenicity for *S. enterica* and *B. subtilis* [22]. These studies were done with the pure compounds; our literature search did not yield any publications on possible degradation products for PEA, PEA-HCl, or EAA.

While determining the best experimental conditions, as well as selecting the graphs for presentation, we performed a number of experiments that are not presented in this Communication. Some of these are included in the Supplementary Materials. The USDA lists 160 F (71.1 °C) as the recommended cooking temperature for ground beef, 145 F (62.8 °C) for steaks and chops, and 165 F (73.9 °C) for all poultry (https://www.foodsafety.gov/food-safety-charts/safe-minimum-cooking-temperature; accessed on 9 April 2021). Starting with this information, we performed a cooking experiment, where ¼ pound burgers were heated in a frying pan. When the inner temperature of the burger had reached 73.9 °C, we measured between 190 F (87.8 °C) and 200 F (93.3 °C) on the outside (see Supplementary Materials Figure S1). It was decided to use 73.9 °C and 93.3 °C to heat our samples.

Among the other decisions that needed to be made was the choice of solvent for the anti-microbials. We performed numerous experiments where either PEA or PEA-HCl and EAA were dissolved in water and present two of these experiments in Figures S2 (PEA-HCl) and S3 (EAA). We never saw a significant difference between heated and unheated samples. From four replicate experiments with PEA-HCl, we calculated an average of 88 µmol mL^−1^ ± 8.4% for the unheated sample, 80 µmol mL^−1^ ± 28.9% for the 73.3 °C sample (10% decrease), and 88 µmol mL^−1^ ± 6.2% for the 93.3 °C sample (no change). From four replicate experiments with EAA, we calculated an average of 66 µmol mL^−1^ ± 24% for the unheated sample, 74 µmol mL^−1^ ± 6.5% for the 73.9 °C sample (11% increase), and 64 µmol mL^−1^ ± 18.3% for the 93.3 °C sample (3.5% decrease).

However, the companies that we contacted during the commercialization process for our technology also questioned whether our anti-microbials might interact with the amino acids that constitute much of the beef meat during the heating process. For this reason, we decided to present the graphs from the data set that was acquired with beef broth in this Communication (Figure 1 and Figure 2). Again, there was no difference in the chromatograms and spectra between heated and unheated samples. A final decision was to be made regarding whether to use PEA or PEA-HCl for the GC–MS experiments. The SDBS database lists GC–MS data for PEA, but not for PEA-HCl. This is because analyzing PEA-HCl will lead to the formation of PEA and HCl. Therefore, the analyzed compound is still PEA, even if PEA-HCl is injected. The only difference between the PEA-HCl and PEA chromatograms was the small secondary peak for PEA-HCl between 10 and 12 min (Figure S2), which interfered with the quantitative analysis. For these reasons, we decided to use the chromatograms and spectrograms for PEA for this Communication. We did, however, provide graphs for PEA-HCl in the online supplement (Figure S2). For the determination of the MIC and MBC, we used PEA-HCl because this is the actual food anti-microbial. Throughout all our extensive experiments, we never saw a difference between heated and unheated samples. 

The GC–MS determined that PEA and EAA did not produce GC peaks in addition to the 2 min (PEA) and 1 min (EAA) main peaks after the compounds were heated (Figure 1 and Figure 2). This implies that no novel compounds were produced during heating from our original compounds. In addition, the size of the GC peaks was not diminished in the heated samples, when quantitively compared to that from the unheated samples. The chemical analysis of PEA and EAA clearly indicates that the two compounds do not change during heating. Heat degradation was also not dependent on the concentration of the anti-microbial or the beef broth. However, we also sought to determine whether the anti-microbial activities of PEA and EAA would be affected by heating. For this reason, the MIC and MBC determinations were performed, which are often used in pre-clinical experiments to characterize anti-microbial agents [23]. Exposing *E. coli* and *S. enterica* to increasing concentrations of PEA-HCl or EAA did not yield any differences between heated and unheated samples either. The growth profiles (Figure 3) were near identical to one another and MIC and MBC were 20.75 mmol mL^−1^ for PEA-HCl for *E. coli* O157:H7 and *S. enterica* serotype Typhimurium, regardless of the heat treatment of the PEA-HCl sample. For EAA, the MICs were 23.4 and 15.6 mmol mL^−1^ for *E. coli* and *S. enterica*, respectively. MBCs were 31.2 mmol mL^−1^ for all EAA samples for *E. coli* and the pattern of percent reductions across all concentrations tested was very similar for the three samples with *S. enterica*. Altogether, our data indicate that PEA, PEA-HCl, and EAA do not change during heating up to 93.3 °C.

## 4. Materials and Methods

### 4.1. Heat Treatment of EAA and PEA (-HCl)

The effect of the heating process on the possible degradation of PEA(-HCl) and EAA was determined chemically (GC–MS). For the microbiological analysis, PEA-HCl was used. For the majority of the GC–MS experiments, solutions were prepared of 83 µmol mL^−1^ of PEA (Sigma Aldrich; Merck KGaA, Darmstadt, Germany) and 77 µmol mL^−1^ of EAA (Alfa Aesar, Ward Hill, MA, USA). The compounds were dissolved in 1% of beef broth bacterial growth medium or water. Bacto Beef Extract (BD Biosciences, San Jose, CA, USA) is derived by an infusion of beef. Per manufacturer, it contains mixed amino acids, minerals, vitamins, nucleotides, and organic acids. For one experiment, we used five times the above indicated concentrations; the PEA solution was prepared at 413 µmol mL^−1^ of PEA-HCl in 5% beef broth, the EAA solution was prepared at 380 µmol mL^−1^ of EAA in 5% of beef broth. For the MIC and MBC determinations, concentrations of PEA-HCl (TCI America, Portland, OR, USA) were prepared in the range of 0 to 88.3 mmol mL^−1^ and EAA in the range of 0 to 31.2 mmol mL^−1^. For MIC and MBC determinations, PEA-HCl and EAA were dissolved in 1% beef broth. Samples were heated to 73.9 °C on a temperature-controlled hotplate. Additional samples were heated to 93.3 °C. For comparison, samples of PEA(-HCl) or EAA were not treated with heat but kept at room temperature.

### 4.2. Analysis by GC and MS 

GC–MS analysis was performed on an Agilent GC–MS instrument (GC model 7890A, MS model 5975C, Santa Clara, CA, USA) equipped with a HP-5ms UI (30 m × 0.25 mm i.d., 0.25 μm) capillary column and helium as the carrier gas. The two compounds were analyzed at the following conditions: 0.3 μL water solutions of the investigated compounds were injected at 175 °C at a split ratio of 50:1. The GC separation method was started at an oven temperature of 100 °C, held for 1 min, and raised to 300 °C at 50 °C/min, where it was held for 5 min. The electron impact ionization (EI) mass spectra for both compounds were obtained at an electron energy of 70 eV and the observed range was set from 30 to 750 amu.

The integration of the peaks of interest for both compounds was calculated based on extracted ion chromatograms for the following characteristic masses: PEA (121 amu) and EAA (130.1 amu). A calibration curve was produced to calculate the area counts as µmol mL^−1^. For this curve, seven concentrations of PEA between 28 and 165 µmol mL^−1^, as well as seven concentrations of EAA between 26 and 154 µmol mL^−1^, were subjected to GC analysis, using the same protocols as for the unheated and heated beef broth samples. The integrals for the peaks were plotted against the molar concentrations. For PEA, the linear curve was calculated at y = 329,322x − 34,086, with a correlation coefficient of R = 0.9881. For EAA, the curve was y = 328,818x ± 3659.1, with a correlation coefficient of R = 0.9974. In four replicates, this curve was used to calculate µmol mL^−1^ concentrations of PEA and EAA. For the more highly concentrated solutions of PEA-HCl and EAA, standard curves were calculated at y = 10^6^x − 337,145, with a correlation coefficient of 0.955 for PEA and y = 3 × 10^6^x − 319,215 at a correlation coefficient of 0.9772 for EAA.

Mass spectrogram peaks for PEA and EAA from our experiments were compared to the Spectral Database for Organic Compounds (SDBS; https://www.library.ucsb.edu/spectral-database-organic-compounds-sdbs; accessed on 9 April 2021).

### 4.3. Antimicrobial Activities

The determination of MIC and MBC served as a microbiological control for the chemistry experiments. For both PEA-HCl and EAA, an unheated control sample was used, as well as the same compounds heat-treated at 73.9 °C and 93.3 °C. Heat treatments were performed as described in Section 4.1. Strains of *E. coli* O157:H7 EDL932 [24] and *Salmonella enterica* serotype Typhimurium FSL R6-0020 (Cornell University) were grown in beef broth at 30 °C overnight, pelleted by centrifugation, and resuspended in liquid beef broth to an OD_600_ of 1.0. Bacterial growth medium was 1% beef broth, supplemented with PEA-HCl (unheated, 73.9 °C, 93.3 °C) at concentrations ranging from 0 to 88.3 mmol mL^−1^, or EAA (unheated, 73.9 °C, 93.3 °C) at concentrations between 0 and 31.2 mmol mL^−1^. Bacteria were inoculated 1:100 into 200 µl of bacterial growth medium on individual wells of a 96-well plate and incubated at 30 °C. Growth was quantified as OD_600_ with a Synergy H1 Hybrid Reader (BioTek Instruments, Inc., Winooski, VT, USA) after 24 h. Averages and standard deviations were determined across four replicates that were produced from cultures grown independently overnight. Data were expressed as OD_600_, with a lower limit of quantitation (LOQ) of 0.01. The lowest concentration at which a “no growth plateau” was reached was considered the MIC for this anti-microbial and bacterium. Using concentrations slightly lower to slightly higher than the MIC, we plated serial dilutions of 24 h cultures onto Luria–Bertani broth (LB; 1% tryptone, 1% NaCl, 0.5% yeast extract) agar plates to calculate bacterial live counts as colony forming units (CFU) per mL. The limit of detection (LOD) was 100 CFU/mL for this experiment. The lowest concentration at which a 99.9% reduction in CFU/mL was observed was considered the MBC for the respective anti-microbial and bacterium.

## 5. Conclusions

This study is significant to the application of our technology because of the potential production of toxic by-products during cooking of the meat. Dissolved in water or beef broth, neither PEA(-HCl) nor EAA produced chromatograms or spectra that were noticeably different between heated and unheated samples. Additionally, the anti-microbial activities of our compounds were not impacted by heating up to 93.3 °C. Our conclusion from this study is that our anti-microbial compounds were not altered by the heating process. This means that the toxicity should not be any higher after heating than it was before.

## 6. Patents

This work did not result in any patents. However, there is a patent pending from research that led to the question addressed by this study. The patent application is listed as US 2019/0082688, published on 21 March 2019.

## Figures and Tables

**Table 1:** Comparison of the experimental and database mass spectra for PEA.

Peak (*m/z*)	30	39	65	91	92	121
**Mass Fragments**	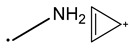		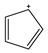	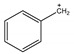	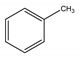	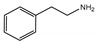
**PEA ^1^**	100	8	18	46	15	13
**SDBS ^2^**	100	3.2	6	15	7	6

^1^ Major peak from the MS that was performed on PEA. ^2^ Comparison with the Spectral Database for Organic Compounds (SDBS). The 3rd and 4th rows show peak abundance (%) for each mass fragment.

**Table 2:** Comparison of the experimental and database mass spectra for EAA.

Peak (*m/z*)	43	60	85	88	130
**Mass Fragments**	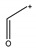	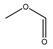	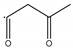	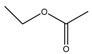	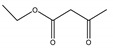
**EAA ^1^**	100	13	27	35	15
**SDBS ^2^**	100	10	15	19	6

^1^ Major peaks (1 min) from the GC that was performed on EAA. ^2^ Database comparison. The 3rd and 4th rows show peak abundance (%) for each mass fragment.

**Table 3:** MBC determination for EAA.

	*E. coli*			*S. enterica*		
EAA (mmol mL^−1^)	Unheated	73.9 °C	93.3 °C	Unheated	73.9 °C	93.3 °C
**0**	0	0	0	0	0	0
**15.6**	98.33	99.72	99.62	99.82	99.75	**99.99**
**23.4**	99.68	99.82	99.85	99	99.77	99.95
**31.2**	**99.99**	**99.99**	**99.99**	**99.99**	**99.99**	99.99
**46.8**	99.99	99.99	99.99	99.99	99.99	99.99

MBCs are expressed as percent reductions, as compared to the samples that did not receive any EAA. A reduction in live bacterial counts of 99.9% was used as cutoff for the MBC. The first concentration at which this reduction was accomplished is printed in bold.

## Data Availability

There are no publicly archived data sets.

## Supplementary Materials

The following are available online at https://www.mdpi.com/article/10.3390/antibiotics10040418/s1, Figure S1: Temperature profile of a burger during cooking, Figure S2: Chromatograms and spectra of PEA-HCl in water, Figure S3: Chromatograms and spectra of EAA in water.
